# Efficacy of a bivalent killed whole-cell cholera vaccine over five years: a re-analysis of a cluster-randomized trial

**DOI:** 10.1186/s12879-018-2981-4

**Published:** 2018-02-20

**Authors:** Youyi Fong, M. Elizabeth Halloran, Jin Kyung Park, Florian Marks, John D. Clemens, Dennis L. Chao

**Affiliations:** 1 Vaccine and Infectious Disease Division, Fred Hutchinson Cancer Research Center, 1100 Fairview Ave N., Seattle, 98109 WA USA; 2 Department of Biostatistics, School of Public Health, University of Washington, Seattle, 98195 WA USA; 3 Epidemiology Unit, International Vaccine Institute, Seoul, Republic of Korea; 4 icddr,b, Dhaka, Bangladesh; 5 Institute for Disease Modeling, Intellectual Ventures, Bellevue, 98005 WA USA; 6 The Department of Medicine, The University of Cambridge, Cambridge, United Kingdom

**Keywords:** Cholera, Cluster randomized trial, Randomized control trial, Vaccination

## Abstract

**Background:**

Oral cholera vaccine (OCV) is a feasible tool to prevent or mitigate cholera outbreaks. A better understanding of the vaccine’s efficacy among different age groups and how rapidly its protection wanes could help guide vaccination policy.

**Methods:**

To estimate the level and duration of OCV efficacy, we re-analyzed data from a previously published cluster-randomized, double-blind, placebo controlled trial with five years of follow-up. We used a Cox proportional hazards model and modeled the potentially time-dependent effect of age categories on both vaccine efficacy and risk of infection in the placebo group. In addition, we investigated the impact of an outbreak period on model estimation.

**Results:**

Vaccine efficacy was 38% (95% CI: -2%,62%) for those vaccinated from ages 1 to under 5 years old, 85% (95% CI: 67%,93%) for those 5 to under 15 years, and 69% (95% CI: 49%,81%) for those vaccinated at ages 15 years and older. Among adult vaccinees, efficacy did not appear to wane during the trial, but there was insufficient data to assess the waning of efficacy among child vaccinees.

**Conclusions:**

Through this re-analysis we were able to detect a statistically significant difference in OCV efficacy when the vaccine was administered to children under 5 years old vs. children 5 years and older. The estimated efficacies are more similar to the previously published analysis based on the first two years of follow-up than the analysis based on all five years.

**Trial registration:**

ClinicalTrials.gov identifier NCT00289224

**Electronic supplementary material:**

The online version of this article (10.1186/s12879-018-2981-4) contains supplementary material, which is available to authorized users.

## Background

An estimated 3 million cholera cases occur each year, about 100,000 of which are fatal [1, 2]. Although the disease is preventable with the provision of clean water and sanitation facilities, this would not be feasible in the near-term in most cholera-endemic regions. A short-term solution may be the use of oral cholera vaccine (OCV). Vietnam regularly uses OCV to control cholera, and other countries with endemic cholera are currently considering this option [3–5]. Improved estimates of the effectiveness of mass vaccination and the duration of protection, thought to be 3 to 5 years [6, 7], would be useful when planning mass OCV deployment in settings with limited resources.

In 2006, over 66,000 people received two doses of either OCV or a placebo in a large cluster-randomized, double-blind, placebo-controlled trial in Kolkata, India [8]. The vaccine was found to be safe and effective at the two-year interim analysis [8], and the vaccine was licensed in India in 2009 and prequalified by the World Health Organization (WHO) in 2011. The final analysis, after 5 years of follow-up, found that the vaccine conferred protection over 5 years, at least among older children and adults [7], but important questions remained unanswered. Rather than waning, protective efficacy appeared to fluctuate with no obvious trend during the 5 years of follow-up, and the study, which was not powered to estimate efficacy by age, did not find statistically significant efficacy differences among age groups.

Here, we re-analyze results from the Kolkata cholera vaccine trial in order to study the efficacy of OCV in different age groups and to look for evidence of waning efficacy over time. Strictly speaking, because of the cluster randomized design of the trial, we are estimating the *total effectiveness* of vaccination, which include the direct and indirect effects [9]. Here, we use the term *vaccine efficacy* for consistency with previous published results from this trial. Analyzing outcomes from a trial with long follow-up presents challenges. Young children in cholera-endemic settings have the highest risk of illness, but their risk should decline as they age, possibly affecting estimates of both cholera risk by age and vaccine efficacy. In addition, a large outbreak occurred in March-April 2010 during the fourth year of the trial [10], which accounted for about one quarter of the total cholera cases during the trial. The outbreak did not affect all geographic regions and age groups equally, which could affect analyses using age and place of residence as covariates. We address these concerns by comparing models that use different assumptions about a vaccinated or unvaccinated individual’s risk of cholera as he or she ages, and by comparing results when including or excluding events that occurred during the large cholera outbreak.

## Methods

### Data

The design and implementation of the OCV trial (ClinicalTrials.gov identifier: NCT00289224) has been described in detail [8]. Briefly, the trial was conducted in three wards of the urban slums of Kolkata, India, with a population of about 109,000. Study clusters were defined by dwellings, each of which comprises one or more households with shared access to water and bathrooms or latrines. Clusters were randomized 1:1 to cholera vaccine or heat-killed *Escherichia coli* K12 placebo stratified by ward of residence (29, 30 and 33) and cluster size (20 or fewer individuals or more than 20 individuals during a pre-study census). Those one year old and older and not pregnant were eligible to participate in the trial. The first vaccine dose was administered between July 27 and August 13, 2006. The second dose was administered between August 27 and September 20, 2006. Only those who received both doses of OCV or placebo were considered in the per-protocol analyses. The per-protocol set contains 31,619 OCV and 34,596 placebo recipients. Rectal swabs were obtained from individuals presenting at local clinics with loose stools and were cultured to detect *Vibrio cholerae*. There were 69 and 219 individuals who had culture-confirmed cholera among participants in the vaccine and treatment arms, respectively, during the five years of follow-up [7]. Three participants had culture-confirmed cholera twice, but only the first episodes were included in the analyses.

### Statistical analysis

**Fig. 1:**
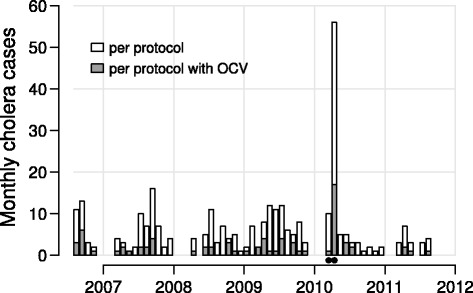
Monthly number of culture-confirmed cholera cases during the study. The number of confirmed cases among all per-protocol study participants are indicated by the bars, with portions shaded gray to indicate the subset randomized to OCV. A large outbreak occurred in March–April 2010, and the bars representing these two months are indicated with black dots

In keeping with previous analyses [7, 8], we analyzed only the first episodes of cholera of each participant and excluded events that occurred less than 14 days after each individual’s second (and final) dose of OCV or placebo. Participants with events within 14 days of their second doses were removed from the analysis. Participants were right-censored when they had cholera, changed households, died, or when the study concluded in 2011. Also in keeping with previous analyses, time to event was treated as the time in days since the participant’s second dose of vaccine or placebo. To study the impact of the large outbreak that occurred in the fourth year of the study (March–April 2010) on the analysis, we also performed analyses in which the participants who are either censored or infected during the outbreak period were excluded from analysis (Fig. 1). The outbreak-free dataset contains 31,393 vaccinees and 34,331 placebo recipients. The distribution of cases by ward and cluster size in the full and outbreak-free data sets are in Additional file 1: Table S1. The distribution of cases by age at baseline and age at risk in the full and outbreak-free data sets are in Additional file 1: Table S2. The distribution of cases by vaccine arm and age group at risk in the full and outbreak-free data sets are in Additional file 1: Table S3. Additional file 2: Figure S1 shows the survival curves stratified by vaccination status and age group at baseline for the full dataset.

To compare different mechanisms that could describe age-dependent OCV efficacy, we tested four Cox models with different age group and treatment interaction. The four models have the same degrees of freedom and contained the same randomization stratification variables (ward of residence and cluster size) as well as additional characteristics of households that were used in the previously published per-protocol analysis [7]: home ownership status, household has a member with a stable occupation, household income is above the median, and household’s distance to the nearest water body is above the median. The four models differ only in how the age groups are defined for the vaccine effect and the natural risk (risk of cholera infection in the placebo arm). The first model (baseline/baseline, or B/B) assumed that both the vaccine efficacy and natural risk were associated with the age group of individuals at baseline (i.e., at the beginning of the trial), the second model (baseline/time-dependent, or B/T) assumed that the vaccine effect was associated with an individual’s age at baseline and the natural risk was associated with the time-dependent age group, the third model (time-dependent/time-dependent, or T/T) assumed that both the vaccine efficacy and natural risk were associated with the time-dependent age group, the last model (time-dependent/baseline, or T/B) assumed that the vaccine effect was associated with time-dependent age group and the natural risk was associated with an individual’s age at baseline. The model equations are in Additional file 3: Supplementary Text. We fit these four models to both the full dataset and an “outbreak-free” dataset that removed subjects with outcomes or censoring events during the major outbreak in 2010.

All Cox models were fitted using the survival package in the R statistical programming system (R Foundation for Statistical Computing; Vienna, Austria) [11]. Cox models with time-dependent age groups were fitted following [12]. To account for cluster randomization, all covariance estimates were robust sandwich estimates [13]. Tests of the proportional hazards assumption in the Cox model were performed using Schoenfeld residuals according to [14] using the identity transformation. The test of overall vaccine efficacy (VE) by age group interaction was performed by a Wald chi-squared test with two degrees of freedom. To plot the time trends of vaccine efficacy, we followed [15] and used a method based on the smoothed scaled Schoenfeld residuals [14]. The VE plots were made using the R function VEplot in the kyotil package, which can be downloaded from the Comprehensive R Archive Network (http://cran.r-project.org/).

To compare the different Cox model fits, likelihood ratio tests cannot be used because the models are not nested. Instead, we used the Bayesian information criterion [16] to help with model choice. Since BIC approximates the Bayes factor, we use the proposed rules for evaluating the strength of evidence in Bayes factors [17] to evaluate the differences in BIC. As the four models all have the same degrees of freedom, the difference in BIC is the same as the difference in twice the log likelihood.

## Results

The only model fits that did not violate the proportional hazards assumption globally were the B/T and T/T models fit to the outbreak-free dataset, while the other six model fits rejected the proportional hazards assumption in the global test with *p* ranging from 0.009 to less than 0.001 (Table 1, Additional file 1: Table S4 and S5). Among the models fit to the outbreak-free dataset, the B/T model has the best likelihood: twice the log likelihood ratio between the B/T and T/T models is 8, and twice the log likelihood ratio between the T/T and B/B models is 25. Thus, the evidence for B/T or T/T against B/B model can be categorized as strong, while the evidence for B/T against T/T can be considered as positive. These results suggest that the most reasonable model fit is the B/T model fit to the outbreak-free dataset.

**Table 1:** Protective efficacy against culture-confirmed cholera of several risk factors

	B/B ^*a*^				B/T ^*b*^				T/T ^*c*^			
	Full ^*d*^		Outbreak-free ^*e*^		Full		Outbreak-free		Full		Outbreak-free	
	1-HR ^*f*^	PH test ^*g*^	1-HR	PH test	1-HR	PH test	1-HR	PH test	1-HR	PH test	1-HR	PH test
Vaccine efficacy												
ages 1 to <5 years	0.45*	0.970	0.38	0.351	0.34	0.250	0.38	0.417	0.47*	0.931	0.44*	0.780
ages 5 to <15 years	0.67**	0.016	0.84**	0.617	0.71**	0.052	0.85**	0.806	0.59**	0.028	0.72**	0.217
ages 15 years and older	0.74**	0.008	0.69**	0.023	0.73**	0.025	0.69**	0.050	0.73**	0.058	0.71**	0.046
Participant age												
1 to <5 years (ref)	0.0		0.0		0.0		0.0		0.0		0.0	
5 to <15 years	0.69**	0.088	0.65**	0.011	0.74**	0.069	0.78**	0.249	0.77**	0.317	0.80**	0.542
15 years and older	0.81**	0.000	0.80**	0.000	0.88**	0.011	0.89**	0.043	0.89**	0.061	0.89**	0.095
Ward of residence												
ward 29 (ref)	0.0		0.0		0.0		0.0		0.0		0.0	
ward 30	0.40**	0.000	0.16	0.102	0.39**	0.000	0.15	0.209	0.39**	0.000	0.15	0.243
ward 33	0.07	0.979	-0.02	0.610	0.07	0.941	-0.01	0.719	0.07	0.755	-0.01	0.751
Large cluster	-0.61	0.382	-0.43	0.026	-0.60	0.403	-0.43	0.023	-0.60	0.384	-0.42	0.025
Own house	0.24	0.189	0.34*	0.549	0.24	0.351	0.34*	0.668	0.24	0.367	0.35*	0.715
Stable occupation	0.27	0.644	0.27	0.539	0.27	0.628	0.27	0.583	0.27	0.527	0.27	0.560
High income	0.32**	0.662	0.38**	0.125	0.32**	0.784	0.38**	0.109	0.32**	0.954	0.38**	0.121
Far from water	-0.31	0.013	-0.09	0.476	-0.31	0.025	-0.08	0.627	-0.31	0.044	-0.09	0.683
Global test ^*h*^		0.000		0.003		0.004		0.312		0.004		0.233
Log lik ratio ^*i*^				106				122				118

^a^both vaccine and age effect are estimated for age group at baseline

^b^vaccine effect is estimated for age group at baseline, age effect is estimated using time-dependent age groups

^c^both vaccine effect and age effect are estimated using time-dependent age groups

^d^all participants were included

^e^participants with events during March–April 2010 were excluded

^f^1-hazard ratio, which can be interpreted as the protective efficacy for the vaccine effect. Significant terms (*p*< 0.05) are marked with * and highly significant terms (*p*< 0.01) are marked with **

^g^*p* from test of proportional hazards (PH) assumption

^h^global test of proportional hazards assumption

^i^log likelihood ratio relative to a Cox model with no covariate

In the B/T model fit to the outbreak-free data, the risk of culture-confirmed cholera infection in the placebo arm was the lowest among adults (those 15 years old and older) and highest among young children (those under 5 years old, see Table 2). Older children (ages 5 to under 15 years) had the highest VE (85%, 95% CI: 67%,93%), followed by adults (69%, 95% CI: 49%,81%), then young children (38%, 95% CI: -2%,62%). The VE for older children was significantly higher than the VE among young children (*p* = 0.002) and also higher, but not significantly, than the VE among adults (*p* = 0.106). Considering the natural age-related risk and VE together, the hazard ratio of a vaccinated older child relative to an unvaccinated young child and that of a vaccinated adult with respect to the same reference group are 0.033 and 0.034, respectively.

**Table 2:** Point estimates and 95% confidence intervals for OCV efficacy by time of vaccination and risk of culture-confirmed cholera by age at time of risk. Estimates are from the B/T model

	Full ^*a*^	Outbreak-free ^*b*^
	1-HR (95% CI) ^*c*^	1-HR (95% CI)
Vaccine efficacy		
ages 1 to <5 years	0.34 (-0.09, 0.60)	0.38 (-0.02, 0.62)
ages 5 to <15 years	0.71 (0.48, 0.84)	0.85 (0.67, 0.93)
ages 15 years and older	0.73 (0.58, 0.83)	0.69 (0.49, 0.81)
Participant age		
ages 1 to <5 years (ref)	0	0
ages 5 to <15 years	0.74 (0.62, 0.83)	0.78 (0.66, 0.86)
ages 15 years and older years	0.88 (0.82, 0.92)	0.89 (0.83, 0.93)

^a^all participants were included

^b^participants with events during March–April 2010 were excluded

^c^1-hazard ratio, which can be interpreted as the protective efficacy for the vaccine effect

To check whether the VE differs by age group, we test the null hypothesis that there is no overall difference between the estimated VE between age groups by a generalized Wald test. The results show that the overall vaccine efficacy by age group interaction was highly significant (*p* = 0.007) in the B/T model fit to the outbreak-free data (Additional file 1: Table S6). In contrast, the VE is not significantly different between age groups (*p* = 0.082) in the B/B model fit to the full dataset, which is consistent with the results from the previous 5-year analysis [7].

Comparing the B/T model fits using the full and outbreak-free datasets, the estimated associated risk of living in ward 30 relative to ward 29 was highly significant (*p* < 0.001) in the fit using the full dataset but not in the fit using the outbreak-free dataset. This was consistent with the fact that ward 29 was most affected by the outbreak and ward 30 was not affected at all (Additional file 1: Table S1). In addition, VE among older children was higher when the model was fit to the outbreak-free dataset than when the full dataset was used.

**Fig. 2:**
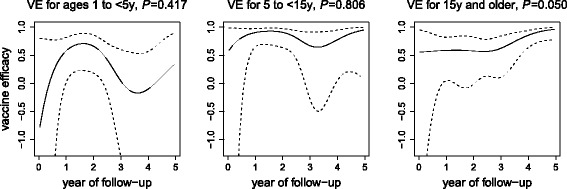
Estimated protective efficacy of OCV over time. Panels plot vaccine efficacy for age groups 1 to under 5 years old (left), 5 to under 15 years old (middle), and 15 years old and older (right). Efficacy estimates are based on model B/T fit to the outbreak-free dataset. 95% point-wise confidence intervals are shown as dashed lines

The estimated VE over time for each age group based on fitting the B/T model to the outbreak-free dataset is shown in Fig. 2. Among adults, there was a trend of increasing VE near the end of the trial (*p* = 0.050 without multi-testing adjustment). Among both young and older children, the VE fluctuated, but the statistical evidence was too weak to conclude anything about trends over time. In addition, the confidence intervals of the estimated VE excluded 0 in year 5 in both older children and adults but not in young children.

## Discussion

This re-analysis of a large Kolkata cholera vaccine trial showed that inclusion or exclusion of the large outbreak in the fourth year of the trial and treating age group as a fixed or time-dependent covariate could have large impacts on the estimated covariate effects on culture-confirmed cholera. The preferred model fit is a hybrid model fit to the outbreak-free data that lets the age group effect be associated with time-dependent age group and the vaccine effect be associated with age group at baseline. Our re-analyses provide additional evidence that the efficacy of OCV varies between different age groups, and suggest that the efficacy among the older children and adults lasted five years in this cholera-endemic setting. We also looked for evidence of waning of efficacy, and we found no evidence, at least among adult vaccinees.

The trial had not originally been powered to estimate differences in VE across age groups, but our re-analysis found more definitive evidence supporting age group-dependent VE than the previous analysis. We found VE to be higher among older children than among young children (*p* = 0.002) or adults (*p* = 0.106). The overall significance is 0.007. The same trend had also been observed in an earlier analysis using only 2 years of follow-up but that result was not statistically significant (overall *p* = 0.07), probably due to the smaller number of events observed [8]. In the previously published analysis using all 5 years of follow-up, adults had the highest estimated VE [7]. The lower VE among older children observed in that study, compared to both the 2-year analysis and ours, can be explained by the inclusion of the March–April 2010 outbreak, which appeared to have disproportionately affected older children (Additional file 1: Table S3). Overall, our age-specific OCV efficacy point estimates of 38%, 85%, and 69% for children under 5 years old, children older than 5 and younger than 15, and individuals 15 years old or older, respectively, look more similar to the published estimates that used the first two years of follow-up (49%, 87%, and 63% [8]) than those using all five years (42%, 68%, and 74% [7]).

The age of participants changed substantially during the 5 years of follow-up. When age enters into a model nonlinearly, e.g., as an age group variable, whether the age is treated as a fixed or time-dependent variable impacts the outcome of the analysis [18]. In most published analyses of clinical trials, age has been treated as fixed. One reason for this may be that the duration of most trials is not long enough to make a material difference to whether or not the age variable is treated as a time-dependent variable. The Kolkata trial analyzed here has 5 years of follow-up and our analyses have demonstrated the importance of treating age group as a time-dependent variable.

An innovative aspect of our analyses is that we decouple the age group that impacts the vaccine effect covariate and the age group that impacts the natural risk. There are four possible combinations: B/B, B/T, T/T and T/B, where the first letter denotes whether VE depends on baseline or time-dependent age group and the second letter denotes whether natural risk depends on baseline or time-dependent age group. *A priori*, it seems biologically more plausible that the natural risk should depend on the time-dependent age group and not on the age at vaccination. This is because as people age, their behavior and life history change and those are likely to impact cholera risk. It also seems more likely that vaccine efficacy depends more on the age group at vaccination than on the time-dependent age group, since the vaccine-elicited immune responses differ by the age at the time of vaccination [19]. Based on these reasonings, the B/T is the most biologically reasonable model while the T/B model is the least. Results from the four fitted models (Table 1 and Additional file 1: Table S4) support the idea that the B/T model is the best model, and the fitted B/T model gives us additional insights over the more conventional B/B model. The estimated hazard ratio between the middle and youngest age group was 0.22 by the B/T model, but the B/B model gave a more attenuated estimate 0.35. This attenuation can be explained by the fact that the younger children group as defined by the B/B model is actually a mixture of the younger and older children at the time of infection.

The occurrence of a major outbreak during follow-up presents an analytical challenge. Our choice of removing the outcomes and censoring events occurring during the outbreak is a simple approach, and it allows us to cleanly assess vaccine efficacy and the effects of other covariates under non-outbreak situations. For example, removing the outbreak from the analysis set results in an upwards shift of the estimated VE among the older children (ages 5 to 15 years) from 0.71 to 0.85. Furthermore, in combination with the use of the B/T model for encoding age group, the evidence for the age group-vaccine treatment interaction grows from borderline (*p*-value 0.082) to highly significant (*p*-value 0.007). It should be noted that vaccinated individuals were protected during the outbreak and exclusion from the analysis does not imply a lack of efficacy during acute outbreaks. Exclusion of the censored and infected subjects during the outbreak has some drawbacks. For example, it is difficult to determine how it may have affected the randomization. We had considered other approaches. One temptation is to use an indicator variable to denote the outbreak period and include the variable in a model to analyze the full dataset. The advantage of this approach is that all data is put to use, but the model is difficult to interpret because the outbreak is not an attribute of the subjects, but rather an outside force that happens at a particular time and place. One may also envision a competing risk model where the events during the outbreak are treated as coming from an alternative cause. As the outbreak only occurs in a relatively short period, there is probably little benefit to take this more complicated approach.

An interesting observation from our analysis is that the cholera risks of a vaccinated older child (ages 5 to 15 years) and a vaccinated adult (15 years old and older) are very close, despite the fact that unvaccinated adults have a lower risk of cholera than an unvaccinated older child. Using young children (ages 1 to 5 years) from the placebo group as reference, the risks experienced by both aforementioned groups are 30-fold lower. This suggests that the vaccine reduced the risk level of those 5 years old and older to a similarly low level.

The results presented here may be relevant for future routine OCV deployment plans in endemic regions where cholera strikes frequently. Our analyses show that OCV could provide 5 years of high-quality protection for those over 5 years old with no evidence of waning among adults, suggesting that they might not need to be re-vaccinated frequently to maintain protection, making mass vaccination more cost-effective in cholera-endemic regions. However, younger children are at high risk of cholera in this population and OCV may confer lower protection, thus re-vaccination when they are older may be desirable.

The duration of protective efficacy of OCV may differ in endemic and epidemic regions. Repeated exposure to *V. cholerae* may serve as natural boosters for vaccinees, prolonging their protection [7, 20]. Therefore, vaccine protection may appear to wane more quickly in populations exposed to cholera less frequently. OCV use outside Asia has largely been to prevent or mitigate outbreaks in populations that do not see cholera every year [21]. OCV was recently used in reactive campaigns during outbreaks in Guinea and Haiti, and the VE estimated during these outbreaks was consistent with the estimates from Kolkata reported here [22, 23], but the duration of protection in these populations is not yet known. Additional studies are needed to estimate how long two doses of OCV can confer protection in populations outside well-studied South Asian endemic regions.

## Conclusions

In this re-analysis of a large placebo controlled trial, we found that oral cholera vaccine efficacy was statistically significantly higher when administered to children 5 years old and older compared to children under 5 years old, a difference that had been suspected but not conclusively demonstrated in previous trials. We also found no evidence of waning vaccine efficacy among adult vaccinees over five years of follow-up, so adults might not require frequent re-vaccination in stable cholera-endemic populations. These findings should be considered when vaccinating endemic populations against cholera.

## Additional files

Additional file 1 **Tables S1–S6.** (PDF 54.8 kb)

Additional file 2 **Figure S1.** (PDF 27.4 kb)

Additional file 3 Supplementary Text (Model equations). (PDF 45.5 kb)

## Abbreviations

CI: Confidence interval
OCV: Oral cholera vaccine
VE: Vaccine efficacy
