# Supplementary material for: Efficacy of a bivalent killed whole-cell cholera vaccine over five years: a re-analysis of a cluster-randomized trial
Source: BMC Infect Dis. 2018 Feb 20;18:84. doi: 10.1186/s12879-018-2981-4 (PMC5819652; doi:10.1186/s12879-018-2981-4)
Supplement: Supplementary file 1 — Tables S1–S6. (PDF 54.8 kb) [file 12879_2018_2981_MOESM1_ESM.pdf]

Table S1. Distribution of the ward of residence and study cluster size of cases during and outside March–April 2010 outbreak.

	Ward			Large cluster	
	29	30	33	No	Yes
Non-outbreak	132 (59%)	38 (17%)	52 (23%)	22 (10%)	200 (90%)
Outbreak	54 (82%)	0 (0%)	12 (18%)	3 (5%)	63 (95%)
Full	186 (65%)	38 (13%)	64 (22%)	25 (9%)	263 (91%)
Total eligible population	30,912 (47%)	15,694 (24%)	19,609 (30%)	13,692 (21%)	52,523 (79%)

Table S2. Distribution of ages of cases at baseline or at risk during and outside the March–April 2010 outbreak.

	Age at baseline (yrs)			Age at risk (yrs)		
	1 to <5y	5 to <15y	15y and older	1 to <5y	5 to <15y	15y and older
Non-outbreak	60 (27%)	58 (26%)	104 (47%)	48 (22%)	65 (29%)	109 (49%)
Outbreak	20 (30%)	22 (33%)	24 (36%)	3 (5%)	35 (53%)	28 (42%)
Full	80 (28%)	80 (28%)	128 (44%)	51 (17%)	100 (35%)	137 (48%)

Table S3. Distribution of ages of cases by OCV vaccination status, during and outside the March–April 2010 outbreak.

	Non-outbreak			Outbreak			Full		
Age at risk (yrs)	1 to <5	5 to <15	15+	1 to <5	5 to <15	15+	1 to <5	5 to <15	15+
Placebo	32	52	87	3	21	24	35	73	111
Vaccine	16	13	22	0	14	4	16	27	26
All	48	65	109	3	35	28	51	100	137

Table S4. Protective efficacy against culture-confirmed cholera of several risk factors - the T/B model: vaccine efficacy is modeled as a function of time-dependent age group while natural risk is modeled as a function of baseline age group.

	T/B			
	Full		Outbreak-	
	PE	PH test	PE	PH test
Vaccine efficacy				
ages 1 to <5 years	0.17	0.421	0.06	0.124
ages 5 to <15 years	0.63**	0.006	0.76**	0.089
ages 15 years and older	0.75**	0.048	0.73**	0.047
Participant age				
5 to <15 years	0.69**	0.043	0.68**	0.040
15 years and older	0.80**	0.000	0.80**	0.000
Ward of residence				
ward 30	0.40**	0.000	0.16	0.150
ward 33	0.07	0.707	-0.01	0.584
Large cluster	-0.61	0.461	-0.43	0.050
Own house	0.24	0.236	0.34*	0.564
Stable occupation	0.28	0.468	0.27	0.482
High income	0.32**	0.973	0.38**	0.151
Far from water	-0.31	0.040	-0.09	0.513
Global test		0.000		0.009
lik ratio				107

Table S5. Point estimates and 95% confidence intervals for OCV efficacy by time of vaccination and risk of culture-confirmed cholera by age at time of risk.

	B/B		B/T		T/T	
	Full	Outbreak-	Full	Outbreak-	Full	Outbreak-
Vaccine efficacy						
ages 1 to <5y	0.45 (0.11, 0.66)	0.38 (-0.03, 0.63)	0.34 (-0.09, 0.60)	0.38 (-0.02, 0.62)	0.47 (0.06, 0.70)	0.44 (-0.01, 0.69)
ages 5 to <15y	0.67 (0.40, 0.82)	0.84 (0.66, 0.93)	0.71 (0.48, 0.84)	0.85 (0.67, 0.93)	0.59 (0.31, 0.76)	0.72 (0.49, 0.85)
ages ≥15y	0.74 (0.58, 0.84)	0.69 (0.50, 0.81)	0.73 (0.58, 0.83)	0.69 (0.49, 0.81)	0.73 (0.58, 0.83)	0.71 (0.53, 0.82)
Participant age						
5 to <15y	0.69 (0.57, 0.77)	0.65 (0.47, 0.76)	0.74 (0.62, 0.83)	0.78 (0.66, 0.86)	0.77 (0.66, 0.85)	0.80 (0.68, 0.87)
≥15y	0.81 (0.72, 0.86)	0.80 (0.70, 0.87)	0.88 (0.82, 0.92)	0.89 (0.83, 0.93)	0.89 (0.83, 0.93)	0.89 (0.83, 0.93)
Ward of residence						
ward 30	0.40 (0.13, 0.58)	0.16 (-0.19, 0.41)	0.39 (0.13, 0.58)	0.15 (-0.21, 0.40)	0.39 (0.12, 0.58)	0.15 (-0.22, 0.40)
ward 33	0.07 (-0.37, 0.37)	-0.02 (-0.50, 0.31)	0.07 (-0.38, 0.37)	-0.01 (-0.49, 0.31)	0.07 (-0.37, 0.37)	-0.01 (-0.50, 0.31)
Large cluster	-0.61 (-1.92, 0.11)	-0.43 (-1.74, 0.25)	-0.60 (-1.91, 0.12)	-0.43 (-1.72, 0.25)	-0.60 (-1.91, 0.12)	-0.42 (-1.71, 0.25)
Own house	0.24 (-0.07, 0.46)	0.34 (0.02, 0.56)	0.24 (-0.07, 0.46)	0.34 (0.03, 0.56)	0.24 (-0.07, 0.46)	0.35 (0.03, 0.56)
Stable occupation	0.27 (-0.10, 0.52)	0.27 (-0.16, 0.54)	0.27 (-0.11, 0.52)	0.27 (-0.16, 0.54)	0.27 (-0.11, 0.52)	0.27 (-0.17, 0.54)
High income	0.32 (0.09, 0.49)	0.38 (0.13, 0.56)	0.32 (0.10, 0.49)	0.38 (0.14, 0.56)	0.32 (0.10, 0.49)	0.38 (0.14, 0.56)
Far from water	-0.31 (-0.74, 0.02)	-0.09 (-0.48, 0.20)	-0.31 (-0.74, 0.02)	-0.08 (-0.47, 0.20)	-0.31 (-0.74, 0.02)	-0.09 (-0.48, 0.20)

Table S6. P-values from testing the overall vaccine efficacy by age group interaction.

	B/B	B/T	T/T
Full	0.082	0.014	0.155
Outbreak-free	0.010	0.007	0.172
