# Supplementary figures and images for: Efficacy of a bivalent killed whole-cell cholera vaccine over five years: a re-analysis of a cluster-randomized trial
Source: BMC Infect Dis. 2018 Feb 20;18:84. doi: 10.1186/s12879-018-2981-4 (PMC5819652; doi:10.1186/s12879-018-2981-4)

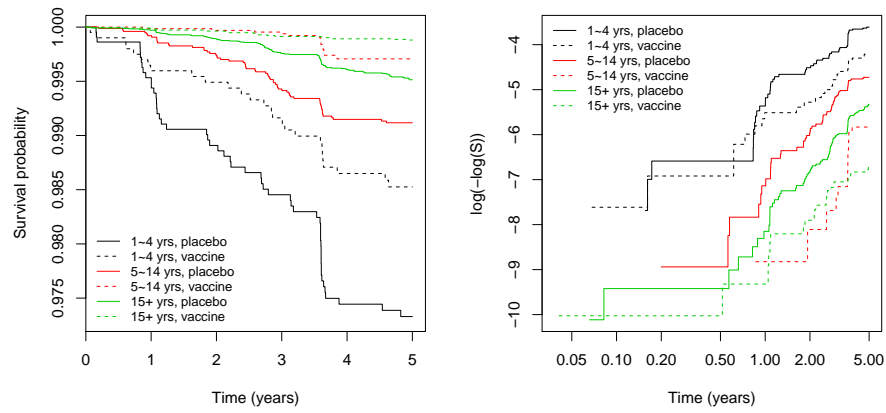

**Figure S1** Survival curves stratified by age group at baseline, full dataset.

Supplement: Supplementary file 2 — Figure S1. (PDF 27.4 kb) [file 12879_2018_2981_MOESM2_ESM.pdf]
