# Supplementary material for: Efficacy of a bivalent killed whole-cell cholera vaccine over five years: a re-analysis of a cluster-randomized trial
Source: BMC Infect Dis. 2018 Feb 20;18:84. doi: 10.1186/s12879-018-2981-4 (PMC5819652; doi:10.1186/s12879-018-2981-4)
Supplement: Supplementary file 3 — Supplementary Text (Model equations). (PDF 45.5 kb) [file 12879_2018_2981_MOESM3_ESM.pdf]

Supplementary Text: Model Equations

B/B: Surv(X,d) ~ trt_young+trt_middle+trt_old+middleagegrp+oldagegrp
+ hh_ward + large_cluster + hh_ownhouse + hh_stableoccupation + hh_hiexpenditure
+ hh_farfromwater + cluster(cl_id)

T/T: Surv(tstart,tstop,d) ~ trt_young +trt_middle +trt_old +middleagegrp+oldagegrp
+ hh_ward + large_cluster + hh_ownhouse + hh_stableoccupation + hh_hiexpenditure
+ hh_farfromwater + cluster(cl_id)

B/T: Surv(tstart,tstop,d) ~ trt_byoung+trt_bmiddle+trt_bold+middleagegrp+oldagegrp
+ hh_ward + large_cluster + hh_ownhouse + hh_stableoccupation + hh_hiexpenditure
+ hh_farfromwater + cluster(cl_id)

T/B: Surv(tstart,tstop,d) ~ trt_young+trt_middle+trt_old+bmiddleagegrp+boldagegrp
+ hh_ward + large_cluster + hh_ownhouse + hh_stableoccupation + hh_hiexpenditure
+ hh_farfromwater + cluster(cl_id)

The formula B/B is used together with the un-augmented dataset, and all the age group-related predictors in the formula are calculated at baseline. The other three formulae are used together with the augmented dataset for time-dependent covariate analysis [1], in which the variables trt_young, trt_middle, trt_old, middleagegrp and oldagegrp are calculated using age group during the time interval defined by tstart and tstop, while the variables trt_byoung, trt_bmiddle, trt_bold, bmiddleagegrp and boldagegrp are calculated using baseline age group.

Reference

[1] Therneau T, Crowson C. Using time dependent covariates and time dependent coefficients in the Cox model. R Survival Package Vignette. 2014.
